# Objective and subjective retrospective evaluation of XERF, a novel single-shot dual-frequency non-invasive monopolar radiofrequency

**DOI:** 10.1007/s10103-026-04996-0

**Published:** 2026-08-18

**Authors:** Guy Erlich, Eliran Dahan, Yoram Wolf

**Affiliations:** 1 The Ruth and Bruce Rappaport Faculty of Medicine, Technion – Israel Institute of Technology, Haifa, Israel; 2 Unit of Plastic Surgery, Hillel Yaffe Medical Center, Hadera, Israel; 3 Energy Based Devices center, G2 Aesthetics, Hadera, Israel

**Keywords:** Monopolar radiofrequency, Skin laxity, Volumetric analysis, Non-invasive skin tightening, Dual-frequency radiofrequency

## Abstract

Facial support is maintained by the vertical anchoring system, a collagen-rich network comprising the dermis, retinacula cutis, superficial musculoaponeurotic system, and retaining ligaments, which progressively deteriorates with age. Noninvasive monopolar radiofrequency (NMRF) induce collagen contraction and remodeling within these structures; however, clinical outcomes with conventional NMRF remain variable. XERF (Cynosure-Lutronic, South Korea) is a dual-frequency NMRF that sequentially delivers 6.78 MHz and 2 MHz energy within a single pulse. This study objectively and subjectively evaluated its efficacy for improving facial skin laxity and skin quality. This retrospective study reviewed adult patients who underwent a single XERF treatment session. Outcomes were extracted from routinely collected clinical data obtained immediately post-treatment and at 1- and 3-month follow-up visits relative to baseline. Objective assessments included three-dimensional stereophotogrammetry for volumetric redistribution, standardized morphometric analysis of periorbital lifting, and computerized skin-quality evaluation. Subjective outcomes were derived from completed prefilled questionnaires, including validated FACE-Q instruments, visual analog scales for perceived age and pain, overall satisfaction, willingness to repeat or recommend treatment, and Patient Global Aesthetic Improvement Scale scores. Safety was assessed through retrospective review of documented adverse events in medical records. Sixteen adult female patients completed follow-up. Objective assessments demonstrated volumetric redistribution, characterized by lateral volume augmentation and medial volume reduction. Periorbital morphometric analysis (*n* = 15) revealed progressive elevation of the eyebrows and upper eyelids. Skin-quality assessment showed improvements in wrinkle severity, skin evenness, pore size, and oiliness, with no significant changes in pigmentation. Subjective outcomes were consistent with objective findings, demonstrating improvements in FACE-Q scores accompanied by a reduction in perceived age. Treatment was well tolerated, with high patient satisfaction and only mild and transient adverse events. In this retrospective study, XERF demonstrates improvements in facial skin laxity and quality, supported by objective and subjective outcomes, with a favorable safety and tolerability profile. These findings should be considered preliminary and require confirmation in larger prospective controlled studies.

## Introduction

Collagen fibers are distributed throughout the dermis [1], retinacula cutis [2, 3], superficial musculoaponeurotic system (SMAS) [4, 5], and retaining ligaments [6, 7], forming an interconnected vertical anchoring system that supports the three-dimensional architecture of the face [2, 3]. With aging, in addition to dermal alterations that contribute to wrinkle formation [1, 8], this cohesive network stiffens and loses its elasticity, leading to disrupted fat compartment organization, diminished structural support, and reduced resistance to gravitational forces, clinically manifesting as sagging and contour irregularities [2, 8].

Noninvasive monopolar radiofrequency (NMRF) generates heat in proportion to the tissue’s intrinsic impedance [9–11], effectively targeting the entire vertical anchoring system [11–13]. Its immediate skin-tightening effect is mediated by collagen contraction, whereas its longer-term clinical benefits result from activation of the wound-healing cascade, leading to tissue remodeling and regeneration [9–11, 14]. This mechanism enables effective treatment with a favorable safety profile and minimal downtime [9, 10, 14].

Different NMRF frequencies produce distinct tissue interaction profiles: higher frequencies concentrate energy within the dermis and the fibrous collagenous structures, while lower frequencies allow deeper penetration with heating of subcutaneous fat [15, 16]. XERF (Cynosure-Lutronic, South Korea) is an advanced NMRF system delivering two frequencies, 6.78 MHz followed by 2 MHz, in a single shot [17–21]. The aim of this study was to objectively and subjectively evaluate the efficacy of XERF for improvement of facial skin laxity and quality.

## Methods

### Ethical considerations

#### Clinical trial number

not applicable.

### Inclusion and exclusion criteria

For this retrospective analysis, medical records of adult patients (≥ 18 years) who underwent a single XERF treatment at the first author’s private clinic in January 2026 were reviewed. Records included standardized clinical photographs and prefilled questionnaires obtained according to routine clinical practice immediately post-procedure and at 1- and 3-month follow-up time points. Records were excluded if patients had undergone any facial aesthetic treatments, including energy-based devices, injectable therapies, or surgical procedures, within 6 months prior to XERF treatment or during the 3-month follow-up period. All patients who met the inclusion criteria and did not meet any exclusion criteria during the study period were included in the analysis.

In accordance with standard clinical practice, all patients did not have known contraindications to NMRF; these contraindications included the presence of implanted electronic devices (e.g., pacemakers or defibrillators), active skin infection or inflammatory dermatoses in the treatment area, pregnancy or breastfeeding, uncontrolled systemic disease, active malignancy in the treatment area, or other conditions deemed by the treating physician to pose a safety risk.

### Treatment Protocol

Each patient underwent a single XERF treatment session. A topical anesthetic cream was not recommended and applied only upon patient request (*n* = 4), containing lidocaine 18% and prilocaine 5% in a white paraffin ointment base (Maayan Haim, Rishon Lezion, Israel), applied 30 min prior to treatment and removed immediately before the procedure. A grounding return pad was placed on the lower left side of patient waist.

A standardized single-session treatment protocol was used for all patients (Table 1). As previous studies have employed single-, two-, and multiple-session regimens, a single treatment was selected to evaluate the minimal treatment effect. Treatment was performed in deep dual-frequency mode (6.78 MHz followed by 2 MHz), with the integrated cooling device (ICD) maintained at level 1 (device range 1–3). Energy levels were individualized according to patient tolerance and titrated to achieve mild to moderate discomfort, corresponding to a pain score of 2–3 on a standardized four-point scale.

**Table 1:** Standardized XERF treatment parameters used for all study participants. Energy levels were individualized according to patient tolerance, while all other treatment parameters were standardized across the study cohort. ICD - Integrated Cooling Device

Parameter	Protocol
Sessions	*N* = 1
Mode	“Deep” (6.78 MHz followed by 2 MHz)
ICD	Level 1
Lower and middle face	
Applicator	Effector 60 (E60; 20 × 30 mm)
Total shots	150 shots per facial side (300 total)
Energy level	Levels 4–7
Treatment technique	50% gliding and 50% stamping
Upper face	
Applicator	Effector 10 (E10; 10 × 10 mm) followed by Effector 5 (E5; 10 × 5 mm)
Total shots	150 shots per facial side (300 total): 100 E10 followed by 50 E5
Energy level	Levels 4–6
Treatment technique	100% stamping

**Fig. 1:**
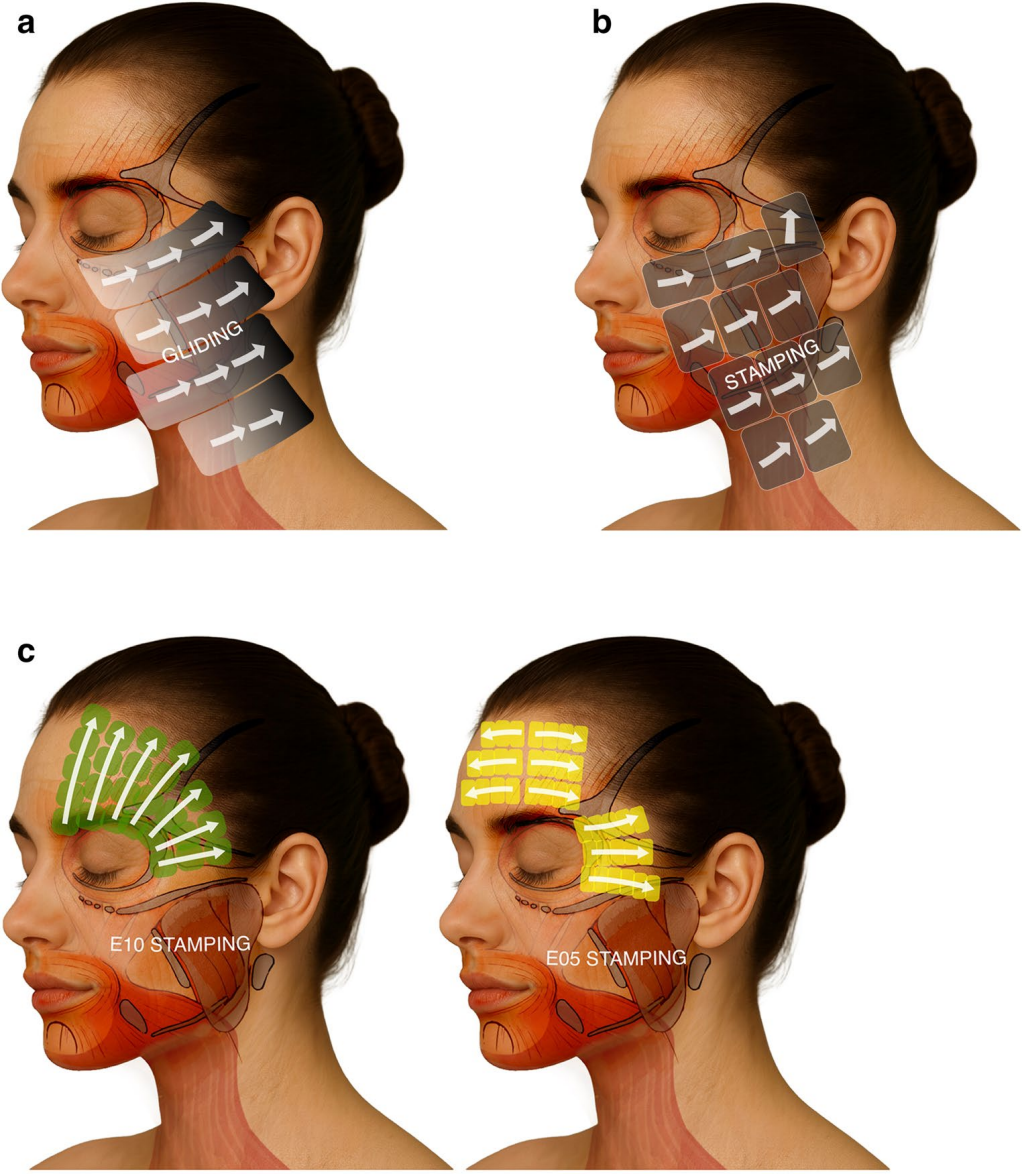
XERF treatment protocol illustrating gliding (**A**) and stamping (**B**) techniques for the lower and middle face, and sequential stamping for the upper face and periorbital region (**C**). (**A**) Continuous gliding passes are performed with the Effector 60 applicator along superolateral vectors across the mandibular, buccal, and lateral facial regions to promote uniform thermal distribution. (**B**) Sequential stamping pulses are then delivered with the Effector 60 applicator along the same vectors to provide targeted dermal and fascial heating. (**C**) Standardized upper-face treatment performed using sequential stamping pulses with the Effector 10 followed by the Effector 5 applicator, applied exclusively to the forehead, temporal, and periorbital regions outside the orbital rim, including the infra-eyebrow region

For the lower and middle face, treatment was delivered using the XERF Effector 60 (E60; 20 × 30 mm). Following impedance synchronization, a total of 150 shots per facial side were delivered at energy levels 4–7 (80–120 J per shot; total energy 24,000–36,000 J for 300 shots). Treatment consisted of alternating gliding preheating and stamping passes, with approximately 50% of the shots delivered in gliding mode and 50% in stamping mode. A single full-face gliding pass (Fig. 1A) was immediately followed by a single full-face stamping pass (Fig. 1B.), and this sequence was repeated throughout the treatment. Gliding was performed in a single direction, as illustrated in Fig. 1A, without overlap between adjacent passes. The XERF system incorporates real-time skin surface temperature monitoring, with automatic interruption of RF delivery if the skin surface temperature exceeds 43 °C. This safety threshold was not reached during any treatment.

For the upper face, treatment was performed using smaller applicators, Effector 10 (E10; 10 × 10 mm) and Effector 5 (E5; 10 × 5 mm) (Fig. 1C.). Following impedance synchronization at the temple, deep-mode was delivered, at energy levels 4–6, exclusively by vector-based stamping, without gliding preheating, to the forehead, temporal, and periorbital regions outside the orbital rim, including the infra-eyebrow region. Gentle upward traction stabilized the upper-eyelid skin to facilitate treatment of the infra-eyebrow area while remaining outside the orbital rim. The applicator was held perpendicular to the skin, with treatment vectors directed laterally and superiorly away from the globe. Adjacent applications were placed without overlap until the predetermined number of shots was delivered. As no energy was applied over the mobile eyelid or toward the globe, and treatment was performed exclusively outside the orbital rim, analogous to routine treatment of the midface immediately inferior to the orbital rim where ocular protection is not required, corneoscleral protective lenses were not used. Effector 10 was used first (100 shots per side) to address brow position and upper-eyelid laxity, followed by Effector 5 (50 shots per side) for targeted treatment of static rhytids [18].

### Outcome Measures

Evaluations were derived from documented time points at baseline, immediately after treatment, and at 1- and 3-month follow-up visits. Outcome measures were categorized into objective imaging-based parameters and subjective patient-reported outcomes.

#### Objective Assessment

Objective assessments were derived from three-dimensional stereophotogrammetry (LifeViz^®^ 3D imaging software, QuantifiCare, France), which provided high-resolution, reproducible analysis from three standardized facial angles. Three-dimensional images were acquired using the LifeViz^®^ Mini stereophotogrammetric system (QuantifiCare, France). Previous studies have demonstrated the suitability of the LifeViz^®^ Mini system for the quantitative assessment of facial soft-tissue changes using standardized stereophotogrammetric imaging protocols [22]. To minimize measurement variability and the inherent measurement error associated with stereophotogrammetry, images acquisition was standardized across all study visits. Patients were instructed to maintain a neutral facial expression while standing in a fixed position, with the head oriented toward a predefined visual target. All images were obtained in the same room under consistent lighting and camera positioning.

**Fig. 2:**
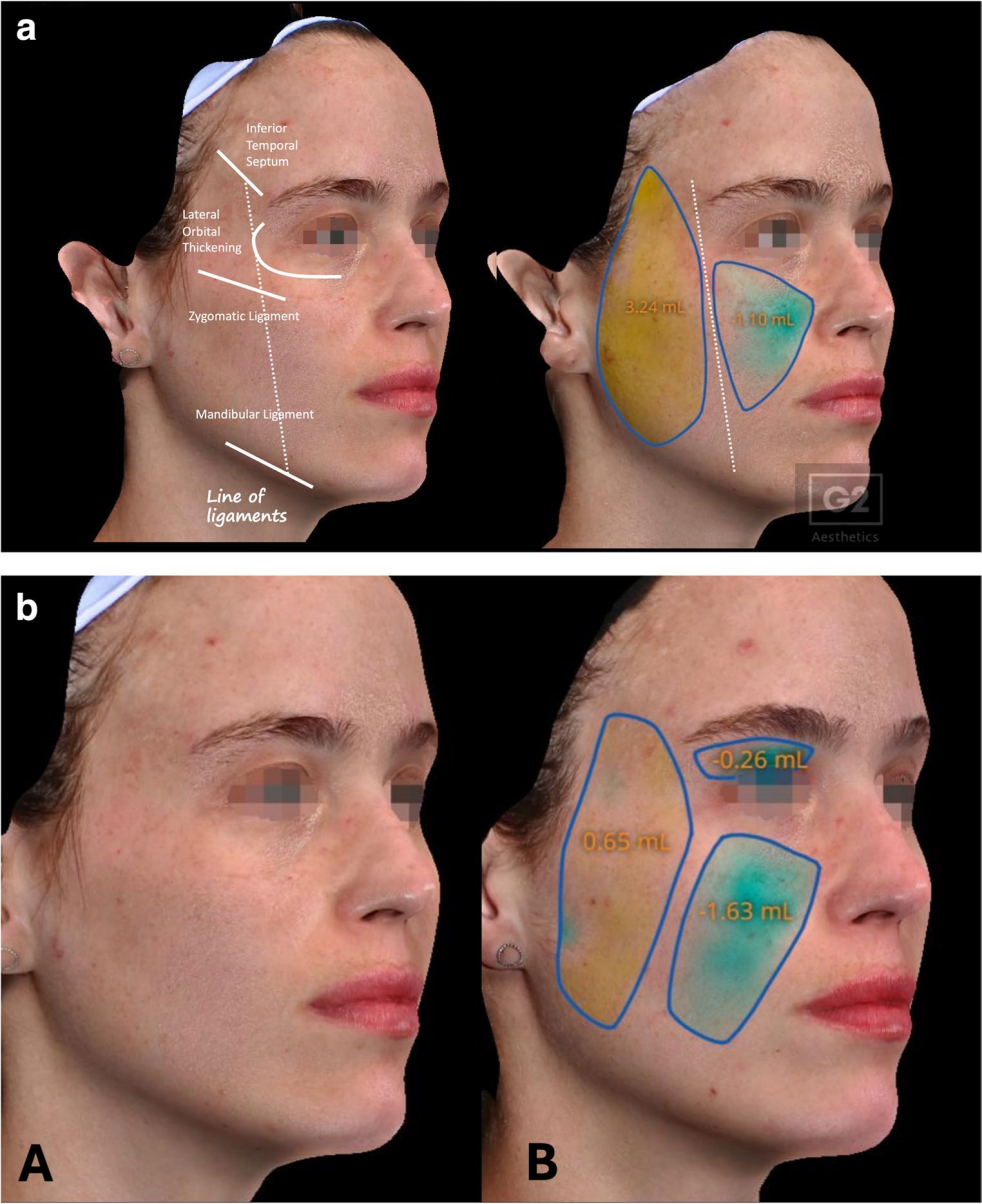
Schematic illustration of the facial retaining ligament framework used to define the volumetric analysis. The dotted white line represents the line of ligaments, connecting the inferior temporal septum, lateral orbital thickening, zygomatic ligament, and mandibular ligament. This anatomical landmark was used to divide the face into medial and lateral compartments for quantitative volumetric assessment. Changes in volume within these predefined compartments were analyzed to explore patterns of relative volumetric redistribution following treatment. Representative LifeViz^®^ stereophotogrammetry outputs illustrating quantitative volumetric changes between baseline and 3-month follow-up in a 29-year-old female. Color-coded maps demonstrate relative regional volume variation within predefined compartments medial and lateral to the “line of ligaments.” Values (mL) represent localized volumetric redistribution patterns explored as an indirect surrogate of tissue tightening and soft-tissue repositioning

Quantitative volumetric evaluation [23, 24] was conducted in predefined medial and lateral facial compartments separated by the “line of ligaments” [25, 26], a continuous anatomical boundary extending from the inferior temporal septum through the lateral orbital thickening, zygomatic ligament, and mandibular ligament (Fig. 2A). As no volumizing intervention was performed, patterns of relative volumetric redistribution characterized by medial compartment volume reduction and gain of volume in lateral compartment, were explored as a potential objective surrogate of tissue tightening and lifting, reflecting possible soft-tissue repositioning (Fig. 2B).

**Fig. 3:**
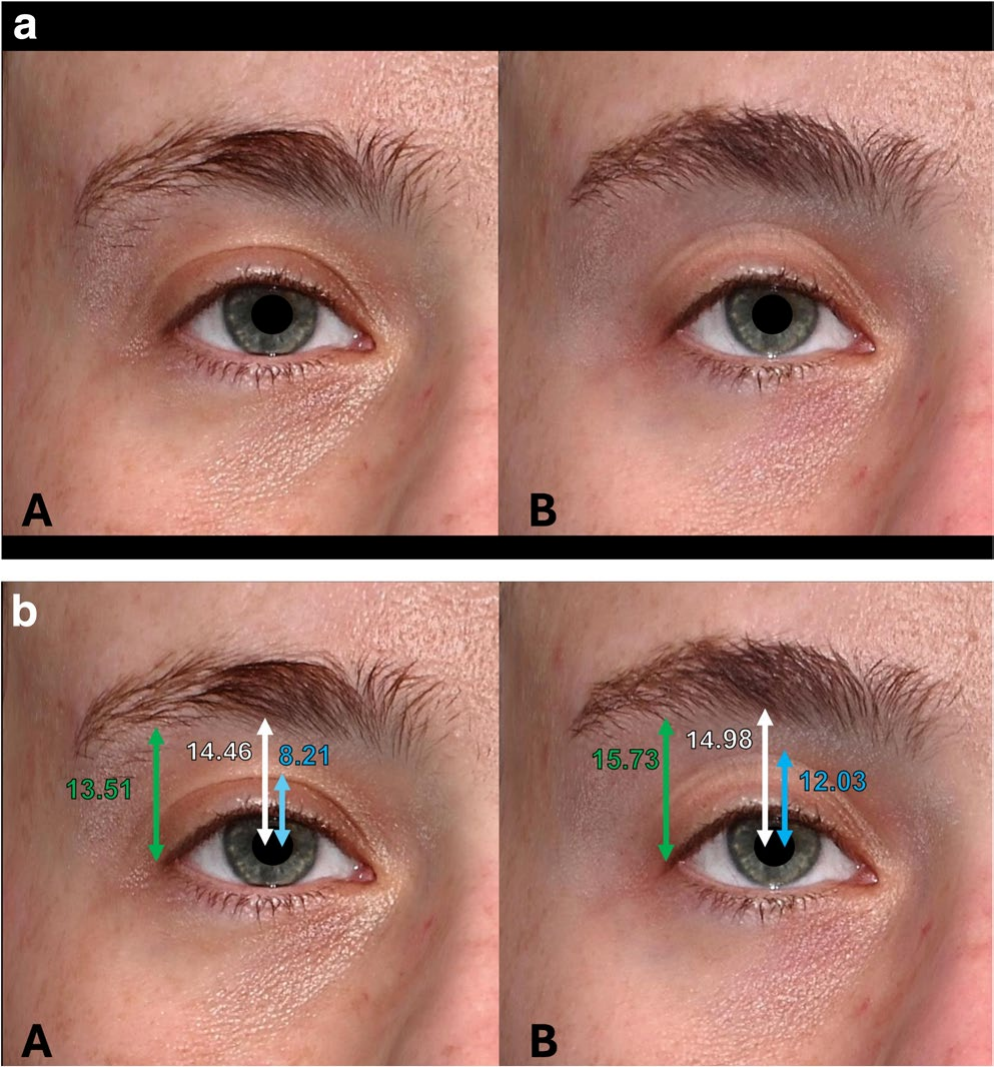
Representative standardized photographs obtained at baseline (**A**) and 3-month follow-up (**B**) in a 29-year-old female following a single XERF treatment session, demonstrating improvement in upper-eyelid laxity and brow position. Representative illustration of the morphometric analysis performed on the images shown in Fig. 3A. Mid-pupillary brow elevation (MPBE), lateral canthal brow elevation (LCBE), and superior palpebral crease elevation (SPCE) were measured as the vertical distances between predefined anatomical landmarks. Increases in these measurements were interpreted as indicative of brow elevation and upper-eyelid tightening

Periorbital lifting was evaluated using standardized linear morphometric measurements reflecting eyebrow position and upper-eyelid contour. Three predefined distances were measured bilaterally. (1) Mid-Pupillary Brow Elevation (MPBE), defined as increase in the vertical distance between the mid-pupillary horizontal reference line and the inferior border of the eyebrow at the mid-pupillary point. (2) Lateral Canthal Brow Elevation (LCBE), defined as increase of vertical distance between the lateral canthal horizontal reference line and the inferior border of the eyebrow at the lateral canthal point. (3) Superior Palpebral Crease Elevation (SPCE), defined as increase in vertical distance between the superior palpebral crease and the central pupillary axis [27]. Due to incomplete eye visualization in one subject, periorbital analysis was performed in 15 participants (*n* = 15). Increases in these measurements were interpreted as indicative of brow elevation and upper-eyelid tightening (Fig. 3A-B).

**Fig. 4:**
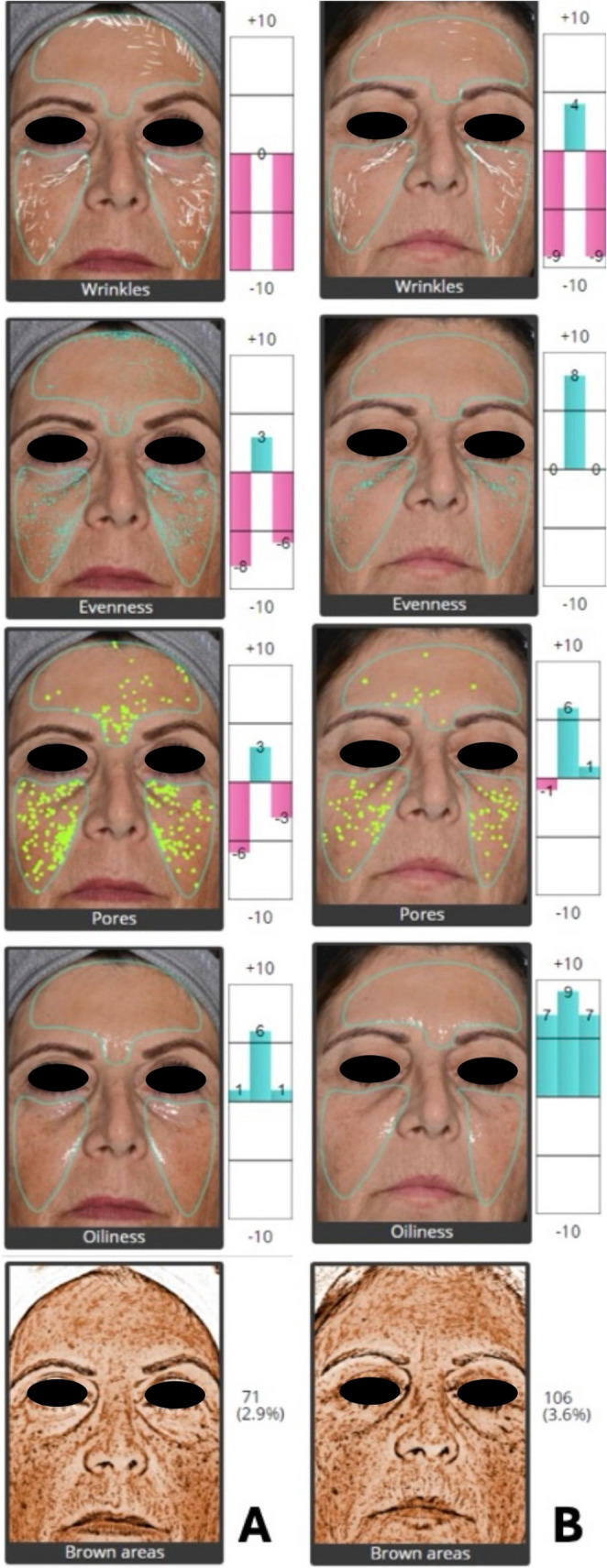
Automated LifeViz^®^ skin quality analysis from standardized frontal imaging of a 66-year-old female. Color-coded maps demonstrate changes in wrinkle severity, skin evenness, pore visibility, and sebaceous activity (oiliness) between baseline (A) and 3-month follow-up (B). Regional scores were normalized to population reference datasets and expressed on a − 10 to + 10 scale. Pigmentation (“brown areas”) was quantified separately as a percentage metric

Objective assessment of skin quality was performed using automated LifeViz® outputs normalized to population reference datasets. Regional scores were generated from standardized frontal imaging across three predefined facial areas (forehead and bilateral cheeks), each expressed on a scale ranging from − 10 to + 10, where negative values indicate worse and positive values indicate better skin status relative to population norms. Regional values were aggregated to derive composite scores (range 0–30) for longitudinal comparison. Pigmentation was reported separately by the software as a percentage metric and was therefore analyzed as an independent continuous variable (Fig. 4).

Adverse events were identified from documented follow-up records, based on recorded patient-reported symptoms and clinical notes.

#### Subjective Assessment

Subjective assessments were based on standardized, prefilled patient-reported outcome measures routinely collected in clinical practice.

Although not specifically validated for NMRF treatments, patient perception of treatment outcomes was assessed using validated Hebrew versions of the FACE-Q instruments [28, 29]. Overall satisfaction with facial appearance was evaluated using the FACE-Q Satisfaction with Facial Appearance module (10 items), assessing perceived facial symmetry, proportionality, freshness, relaxation, appearance in photographs, profile view, and appearance under varying daily conditions. Responses were recorded using a 4-point Likert scale (1 = definitely disagree, 2 = somewhat disagree, 3 = somewhat agree, and 4 = definitely agree), with higher scores indicating greater satisfaction or more positive age perception. Perceived facial aging was evaluated using the FACE-Q Aging Appraisal module (7 items), assessing agreement with statements related to perceived age discrepancy, emotional response to aging appearance, and dissatisfaction with age-related facial changes. Responses were assessed using a four-point Likert scale (1 = definitely disagree, 2 = somewhat disagree, 3 = somewhat agree, and 4 = definitely agree), with higher scores indicating a more negative perception of facial aging (i.e., feeling or appearing older).

Perceived facial age was additionally assessed using a FACE-Q visual analog scale (VAS) ranging from − 15 years (appearing younger than chronological age) to + 15 years (appearing older).

Participants rated overall satisfaction with treatment outcomes using a 5-point Likert scale (1 = very dissatisfied; 5 = very satisfied). Patients were also asked whether they would recommend the treatment to others and whether they would be willing to undergo repeat treatment sessions. Responses were recorded as binary categorical variables (Yes/No).

Patient Global Aesthetic Improvement Scale (PGAIS) assessments were assessed for two domains. (1) Skin texture, fine lines, and pore appearance; and (2) skin tightening and lifting. Each domain was graded on a 5-point scale (1 = worsening, 2 = no improvement, 3 = mild improvement, 4 = significant improvement, and 5 = exceptional improvement).

Procedural discomfort was evaluated immediately after treatment using a separate 11-point visual analog scale (VAS; 0–10), where 0 indicated no pain and 10 indicated unbearable pain. For descriptive interpretation, scores were categorized as no to mild pain (0–3), moderate pain [4–6], and severe pain [7–10].

### Statistical Analysis

All statistical analyses were performed using GraphPad Prism version 11 (GraphPad Software, San Diego, CA, USA). Statistical analyses were performed using parametric or non-parametric tests as appropriate, with significance set at a two-sided α = 0.05. Continuous variables are presented as mean ± SD. Normality was assessed using the Shapiro–Wilk test. For volumetric redistribution, changes from baseline within each compartment (medial and lateral, right and left) were evaluated using one-sample t-tests or Wilcoxon signed-rank tests, depending on the normality of the data. Pairwise comparisons between time points (Day 0 vs. 1 month, Day 0 vs. 3 months, and 1 month vs. 3 months) were performed using paired t-tests or Wilcoxon signed-rank tests, as appropriate. No formal correction for multiple comparisons was applied; given the exploratory and hypothesis-generating nature of this analysis, and the small sample size, results are interpreted accordingly with emphasis on effect magnitude and clinical consistency across compartments rather than individual p-values alone. To evaluate the findings in the context of multiple comparisons, a post hoc sensitivity analysis using the Benjamini–Hochberg false discovery rate procedure was performed. All primary findings remained statistically significant after correction, supporting the robustness of the observed treatment effects.

For periorbital morphometric parameters (MPBE, LCBE, SPCE), temporal changes were evaluated using repeated-measures ANOVA (RM-ANOVA), with Greenhouse–Geisser correction applied when sphericity was violated (LCBE–Left). For skin quality outcomes, RM-ANOVA (evenness, pore size, oiliness) or Friedman tests (wrinkles, pigmentation) were used for overall temporal assessment, with pairwise comparisons conducted via paired t-tests or Wilcoxon signed-rank tests as appropriate. For subjective outcomes, FACE-Q domain scores and perceived age VAS were analyzed using paired t-tests (normality confirmed by Shapiro–Wilk) or Wilcoxon signed-rank tests. Overall temporal effects were assessed using RM-ANOVA or Friedman tests. Effect sizes were reported as Cohen’s d, rank-based r, or partial eta squared (η²p), as appropriate. Categorical variables are presented as frequencies and percentages. Significance levels were defined as * *p* < 0.05, ** *p* < 0.01, *** *p* < 0.001.

## Results

This retrospective study included 16 adult female patients with a median age of 54.5 years (range 25–73), Fitzpatrick skin phototypes II–V, and varying degrees of facial laxity ranging from mild to severe (Class 1–7 according to the Facial Laxity Rating Scale) [30] (Table 2.). All participants sought facial rejuvenation, underwent a single XERF treatment session, and completed evaluations at baseline, immediately post-treatment, and at 1-month (1 M) and 3-month (3 M) follow-up visits.

**Table 2:** Baseline demographic and clinical characteristics of the 16 patients included in this retrospective study. Age is presented as the median with range

Characteristic	Value
Number of patients	16
Sex	Female, *n* = 16 (100%)
Age, years	Median 54.5 [Range 25–73]
Fitzpatrick skin phototype	II–V
Facial Laxity Rating Scale	Classes 1–7 (mild–severe)

Treatment was well tolerated across all participants. The mean procedural pain score was 4.31 ± 1.71 on the VAS, corresponding predominantly to mild-to-moderate discomfort. Mild pain (VAS ≤ 3) was reported in 38% of patients, moderate pain in 50%, and severe pain (VAS ≥ 7) in 12% (Table 3).

**Table 3:** Procedural pain distribution across the study cohort. Values are presented as mean ± SD, median [IQR], and range. Pain severity categories are defined based on VAS thresholds

Statistic	Value
Mean ± SD	4.31 ± 1.71
Median [IQR]	4.00 [3.00–5.25]
Range (min–max)	2.0–8.0
Mild pain (≤ 3) / 16	6 (38%)
Moderate (4–6) / 16	8 (50%)
Severe pain (≥ 7) / 16	2 (12%) — 1 patient scored 8, 1 scored 7

Only mild and transient adverse events were observed. One patient experienced self-limited erythema lasting one day, another reported erythema persisting for two days, and one patient developed transient erythema accompanied by mild edema that resolved within one day. No cases of thermal injury, persistent complications, or clinical/imaging evidence of fat atrophy were observed.

### Objective Outcomes

#### Volumetric Redistribution

Volumetric outcomes are presented as changes (Δ mL) relative to the baseline stereophotogrammetric image, which served as the reference for all analyses. Positive values indicate a relative increase in compartment volume, whereas negative values indicate a relative decrease.

Immediately post-treatment, the combined lateral compartments (R + L mean) demonstrated a statistically significant increase in volume (+ 1.37 ± 0.99 mL, *p* < 0.001), whereas the medial compartments exhibited a significant reduction (− 0.49 ± 0.56 mL, *p* < 0.01) relative to baseline. This bidirectional redistribution pattern was maintained at 1 month, with a persistent increase in lateral volume (+ 0.49 ± 0.99 mL, *p* < 0.05) and a further reduction in medial volume (− 0.63 ± 0.59 mL, *p* < 0.001). At 3 months, medial volume reduction remained statistically significant (− 0.57 ± 0.56 mL, *p* < 0.01), whereas lateral changes were no longer significant at the group level (Table 4).

**Table 4:** Volumetric changes (mL) from baseline across medial and lateral facial compartments at each time point. Values represent mean ± SD. Asterisks denote statistical significance versus baseline (one-sample t-test or Wilcoxon signed-rank test). All values represent changes from baseline; baseline is set as 0 (reference) for all compartments

Compartment	Immediately After (Mean ± SD)	1 M (Mean ± SD)	3 M (Mean ± SD)
Lateral (R + L mean)	+ 1.37 ± 0.99 ***	+ 0.49 ± 0.99 *	+ 0.47 ± 1.13 ns
Medial (R + L mean)	−0.49 ± 0.56 **	−0.63 ± 0.59 ***	−0.57 ± 0.56 **
Lateral — Right	+ 1.32 ± 1.18 ***	+ 0.13 ± 0.94 ns	+ 0.39 ± 1.37 ns
Lateral — Left	+ 1.43 ± 1.38 ***	+ 0.84 ± 1.40 **	+ 0.54 ± 1.19 ns
Medial — Right	−0.63 ± 0.71 **	−0.76 ± 0.73 ***	−0.70 ± 0.58 ***
Medial — Left	−0.34 ± 0.63 *	−0.50 ± 0.57 **	−0.43 ± 0.75 *

In the lateral compartments, both the right and left sides exhibited significant immediate post-treatment volume increases (right: +1.32 ± 1.18 mL, *p* < 0.001; left: +1.43 ± 1.38 mL, *p* < 0.001). However, the persistence of this effect differed between sides, with the left lateral compartment maintaining a significant increase at 1 month (+ 0.84 ± 1.40 mL, *p* < 0.01), while the right side did not demonstrate a statistically significant change at this timepoint (+ 0.13 ± 0.94 mL, ns). By 3 months, neither side showed significant lateral volume gain (Table 4.)

In contrast, medial compartments demonstrated a more stable and sustained response. The right medial compartment exhibited consistent and statistically significant volume reduction across all timepoints (Immediate after: −0.63 ± 0.71 mL, *p* < 0.01; 1 month: −0.76 ± 0.73 mL, *p* < 0.001; 3 months: −0.70 ± 0.58 mL, *p* < 0.001). The left medial compartment also showed significant reductions at Immediate after and 1 month (− 0.34 ± 0.63 mL, *p* < 0.05; −0.50 ± 0.57 mL, *p* < 0.01, respectively), with a trend toward persistence at 3 months (− 0.43 ± 0.75 mL, *p* < 0.05), albeit with greater variability (Table 4).

Pairwise comparisons demonstrated a significant decrease in lateral volume between Immediate after and 1 month (R + L mean: p = 0.002), followed by stabilization between 1 and 3 months (p = 0.957). In contrast, medial compartments did not exhibit significant differences between timepoints, indicating that the reduction in medial volume achieved immediately post-treatment was maintained throughout the follow-up period (Table 5).

**Table 5:** Pairwise comparisons of volumetric changes between time points across facial compartments. P-values represent comparisons between specified time intervals

Compartment	Immediately After vs. 1 M	Immediately After vs. 3 M	1 M vs. 3 M
Lateral (R + L mean)	0.002 **	0.022 *	0.957 ns
Medial (R + L mean)	0.134 ns	0.640 ns	0.747 ns
Lateral — Right	0.004 **	0.038 *	0.521 ns
Lateral — Left	0.028 *	0.060 ns	0.465 ns
Medial — Right	0.148 ns	0.703 ns	0.799 ns
Medial — Left	0.387 ns	0.711 ns	0.779 ns

The combined medial and lateral volumetric changes at 3 months showed no statistically significant net change in facial volume on either side. Mean changes were − 0.31 ± 1.64 mL on the right and + 0.11 ± 1.66 mL on the left (Table 6)

**Table 6:** Net volumetric change per hemifacial unit (combined medial and lateral compartments) at 3 months showed no significant deviation from zero on either side. confidence interval; SD, standard deviation; ns, not significant

	Mean ± SD	95% CI	*p*
Right	−0.31 ± 1.64 mL	[− 1.19, + 0.57]	*p = 0.462 ns*
Left	+ 0.11 ± 1.66 mL	[− 0.78, + 0.99]	*p* = 0.801 ns

#### Periorbital and Brow Elevation

Periorbital analysis was performed in 15 patients, as one participant was excluded due to incomplete eye visualization at one timepoint. Morphometric analysis demonstrated progressive and time-dependent improvements in periorbital lifting parameters across the evaluated follow-up period.

Mid-pupillary brow elevation (MPBE) increased gradually over time, from a baseline mean of 14.01 ± 2.34 mm to 14.35 ± 2.41 mm at Immediate after, 14.86 ± 2.44 mm at 1 month, and 15.12 ± 2.42 mm at 3 months. This change was statistically significant for the combined R + L mean (*p* = 0.023) and for the right side (*p* = 0.038), while the left side demonstrated a non-significant trend (*p* = 0.055) (Table 7).

**Table 7:** Periorbital morphometric measurements (mm) at each time point, including mid-pupillary brow elevation (MPBE), lateral canthal brow elevation (LCBE), and superior palpebral crease elevation (SPCE). Values are presented as mean ± SD. Statistical analysis of time-dependent changes in periorbital measurements using repeated-measures ANOVA (RM-ANOVA). P-values indicate overall temporal effects

Variable	Baseline	Immediately After	1 M	3 M	*p*-value
MPBE — R + L mean	14.01 ± 2.34	14.35 ± 2.41	14.86 ± 2.44	15.12 ± 2.42	0.023^*^
MPBE — Right	13.96 ± 2.64	14.08 ± 2.91	15.06 ± 2.68	14.97 ± 2.57	0.038^*^
MPBE — Left	14.06 ± 2.44	14.62 ± 2.23	14.67 ± 2.50	15.28 ± 2.61	0.055^ns^
LCBE — R + L mean	15.55 ± 1.98	16.69 ± 2.40	16.35 ± 2.54	16.99 ± 2.43	0.009^**^
LCBE — Right	15.15 ± 1.99	15.72 ± 1.87	15.75 ± 2.50	15.81 ± 2.50	0.562^ns^
LCBE — Left	15.95 ± 2.25	17.67 ± 3.03	16.96 ± 2.87	18.16 ± 2.64	0.001^***^
SPCE — R + L mean	8.92 ± 1.86	9.39 ± 2.18	10.01 ± 2.01	10.33 ± 1.93	< 0.001^***^
SPCE — Right	8.85 ± 1.99	8.99 ± 2.12	10.08 ± 2.15	10.17 ± 2.17	< 0.001^***^
SPCE — Left	8.99 ± 1.84	9.80 ± 2.50	9.95 ± 1.99	10.50 ± 1.76	0.005^**^

Lateral canthal brow elevation (LCBE) also demonstrated time-dependent changes, increasing from 15.55 ± 1.98 mm at baseline to 16.69 ± 2.40 mm at Immediate after, 16.35 ± 2.54 mm at 1 month, and 16.99 ± 2.43 mm at 3 months. These changes were statistically significant for the R + L mean (*p* = 0.009) and for the left side (*p* = 0.001), whereas the right side did not show a significant effect (*p* = 0.562) (Table 7).

Superior palpebral crease elevation (SPCE) exhibited the most pronounced and consistent temporal improvement, increasing from 8.92 ± 1.86 mm at baseline to 9.39 ± 2.18 mm atImmediate after, 10.01 ± 2.01 mm at 1 month, and 10.33 ± 1.93 mm at 3 months. These changes were statistically significant across all measurements, including the R + L mean (*p* < 0.001), right side (*p* < 0.001), and left side (*p* = 0.005) (Table 7).

#### Skin Quality (LifeViz^®^ Analysis)

Objective skin-quality analysis demonstrated progressive improvements across multiple parameters over the 3-month follow-up. Wrinkle scores showed a delayed but significant improvement at 3 months compared to baseline (*p* = 0.008), with no significant change at 1 month (*p* = 0.344). Skin evenness improved significantly at 3 months (*p* < 0.001), with continued improvement between 1 and 3 months (*p* = 0.014), indicating progressive enhancement over time. Pore size demonstrated significant improvement at both 1 month (*p* = 0.021) and 3 months (*p* < 0.001), although no further significant change was observed between these timepoints. Oiliness showed a significant reduction at 3 months (*p* = 0.008), with no significant change at 1 month. In contrast, pigmentation did not demonstrate statistically significant changes at any timepoint (all *p* > 0.15). Repeated-measures analysis confirmed significant overall temporal effects for wrinkles (*p* = 0.0059), evenness (*p* = 0.0003), pore size (*p* = 0.0020), and oiliness (*p* = 0.0113), but not for pigmentation (*p* = 0.442) (Table 8).

**Table 8:** Objective skin-quality outcomes derived from LifeViz^®^ imaging. Values are presented as mean ± SD and percentage change from baseline. Pairwise comparisons between time points were performed using paired t-tests or Wilcoxon signed-rank tests, according to data distribution. Longitudinal changes over time were analyzed using repeated-measures ANOVA (RM-ANOVA) or the Friedman test, as appropriate

Parameter	Baseline	1 M	3 M	*P*
Wrinkles	-0.38 ± 12.58	0.94 ± 12.98	3.50 ± 11.70	0.006**
Evenness	13.38 ± 10.83	15.50 ± 10.70	18.63 ± 9.13	0.0003***
Pore	16.19 ± 8.86	18.25 ± 9.45	20.00 ± 7.28	0.002**
Oiliness	14.63 ± 7.24	16.25 ± 6.58	19.19 ± 6.21	0.011*
Pigmentation	2.97 ± 1.25	3.38 ± 1.15	3.48 ± 0.97	0.442ns

### Subjective Outcomes

#### FACE-Q and Perceived Age (VAS)

Patient-reported outcomes were assessed using validated FACE-Q instruments, comprising a total of 17 items per participant (10 items for the Satisfaction with Facial Appearance module and 7 items for the Aging Appraisal module). Both FACE-Q domains demonstrated statistically significant improvements following treatment. Facial satisfaction scores increased significantly from baseline to 1 month (Rasch score: +15.3 points, *p* = 0.004, d = 0.86) and remained significantly elevated at 3 months (*p* = 0.008, d = 0.76). Similar results were observed for raw summed scores, confirming consistency across scoring methods. Aging appraisal scores also showed significant improvement, with Rasch scores increasing by + 16.3 points at 1 month (*p* = 0.003, d = 0.88) and remaining significantly improved at 3 months (+ 10.7 points vs. baseline, *p* = 0.006, d = 0.81), reflecting a reduction in perceived age-related dissatisfaction. No statistically significant differences were observed between 1 and 3 months for either domain (all *p* > 0.15), indicating that the treatment effect was established early and maintained over time without further significant change (Table 9).

**Table 9:** Patient-reported outcomes (FACE-Q and VAS Age): means and paired comparisons. Mean ± SD; *n* = 16. p shown in pairwise columns. FACE-Q tests: paired t-test throughout (all paired differences passed Shapiro–Wilk, *p* ≥ 0.097). VAS Age tests: paired t-test throughout. VAS Age omnibus: RM-ANOVA F [2, 30] = 8.05, *p* = 0.002. FACE-Q omnibus: Satisfaction RM-ANOVA F [2, 30] = 9.10, *p* < 0.001; Aging Friedman χ² [2] = 6.58, *p* = 0.037

Sub-scale	Baseline	1 M	3 M	Bassline vs. 1 M (*p*)	Baseline vs. 3 M (*p*)	1 M vs. 3 M (*p*)
FACE-Q Satisfaction (Rasch 0–100)	48.75 ± 17.79	64.06 ± 17.22	61.00 ± 18.80	0.004**	0.008**	0.277ns
FACE-Q Aging Appraisal (Rasch 0–100)	62.25 ± 24.46	78.56 ± 22.76	72.94 ± 21.84	0.003**	0.006**	0.212ns
VAS Age (years vs. chronological age)	-1.00 ± 4.41	-3.81 ± 5.49	-4.28 ± 5.29	0.018*	0.002**	0.515ns

Patients reported a significant reduction in perceived facial age at both follow-up timepoints. At 1 month, perceived age decreased by − 2.81 years (*p* = 0.018), and at 3 months by − 3.28 years (*p* = 0.002), representing moderate-to-large and large effect sizes, respectively. No significant difference was observed between 1 and 3 months (*p* = 0.515), indicating stabilization of the effect after the first month (Table 9).

#### Patient Satisfaction

Overall satisfaction was high at both timepoints, with mean scores of 4.25 ± 0.68 at 1 month and 4.19 ± 0.83 at 3 months (on a 5-point Likert scale, where 1 = very dissatisfied and 5 = very satisfied), with no significant change over time (*p* = 0.739) (Table 10.). All patients (100%) reported willingness to repeat the procedure and recommend it to others at both follow-up visits (Table 10.)

**Table 10:** Overall patient satisfaction with treatment outcomes (Q1: “How satisfied are you with the results of the treatment?”), assessed on a 5-point Likert scale (1 = very dissatisfied; 5 = very satisfied). Values are presented as mean ± SD, with statistical comparison between time points. Patient-reported willingness outcomes: Q2 (“Would you be willing to repeat the treatment?”) and Q3 (“Would you recommend this treatment to others?”), recorded as binary responses (Yes/No) and presented as frequencies and percentages

Item	1 M	3 M
Satisfaction Q1 (1–5 Likert)	4.25 ± 0.68	4.19 ± 0.83
Satisfaction Q2 (willingness to repeat)	16 / 16 (100%)	16 / 16 (100%)
Satisfaction Q3 (willingness to recommend)	16 / 16 (100%)	16 / 16 (100%)

#### Patient Global Aesthetic Improvement Scale (PGAIS)

PGAIS scores demonstrated improvements across both evaluated domains (skin quality and tightening) 3 months after the treatment (Table 11.).

**Table 11:** Patient Global Aesthetic Improvement Scale (PGAIS) outcomes: Q1 (skin texture, fine lines, and pore appearance) and Q2 (skin tightening and lifting), each assessed on a 5-point scale (1 = worsening; 5 = exceptional improvement). Values are presented as mean ± SD

Item	3 M (mean ± SD)
PGAIS Q1	3.13 ± 0.89
PGAIS Q2	3.31 ± 0.87

## Discussion

**Fig. 5:**
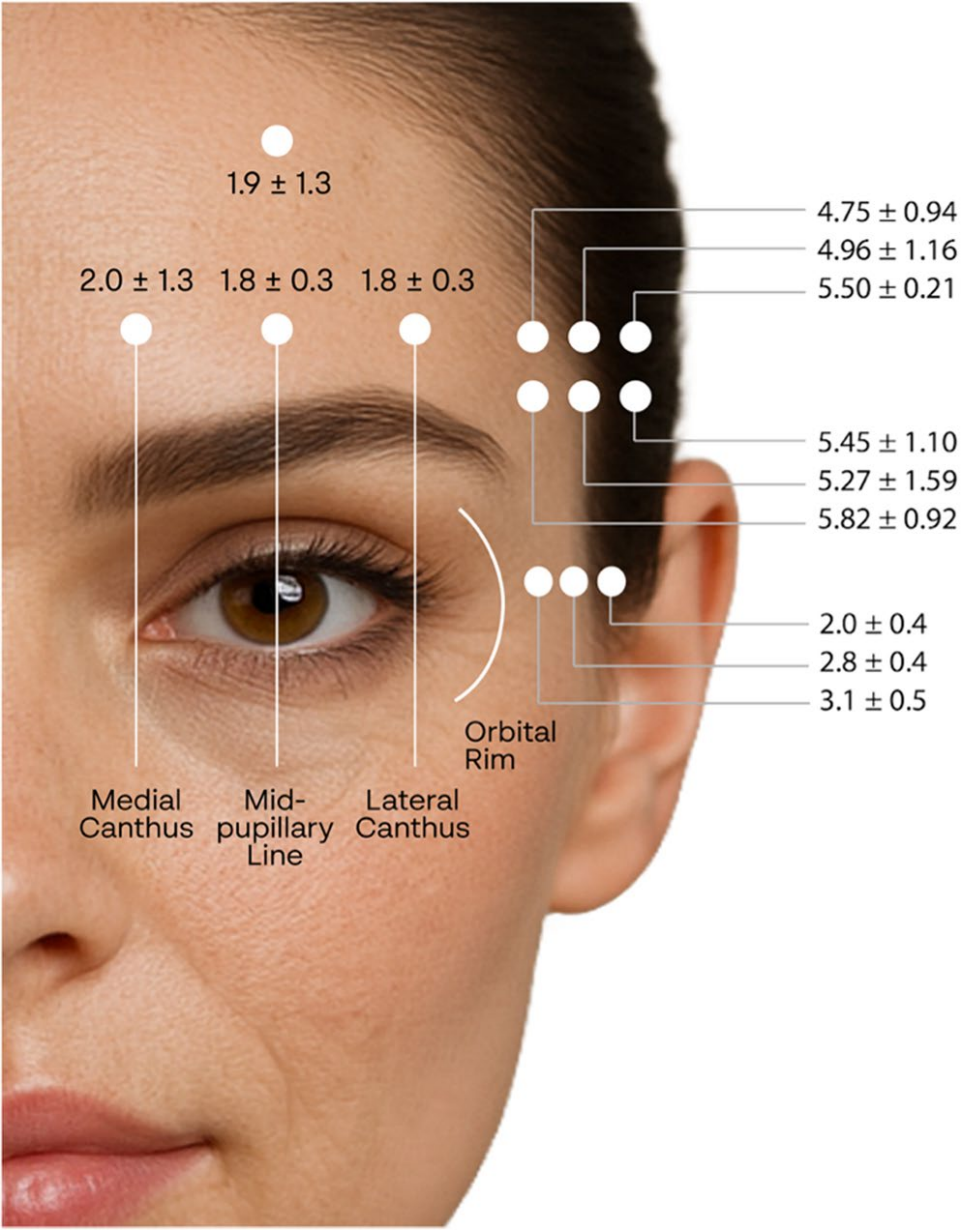
Schematic representation of periorbital SMAS-related depth measurements, including the frontalis muscle, orbicularis oculi muscle, and superficial temporal fascia, assessed by ultrasound relative to key anatomical landmarks. Depth measurements are reported in millimeters, providing both the median values and their corresponding ranges

**Fig. 6:**
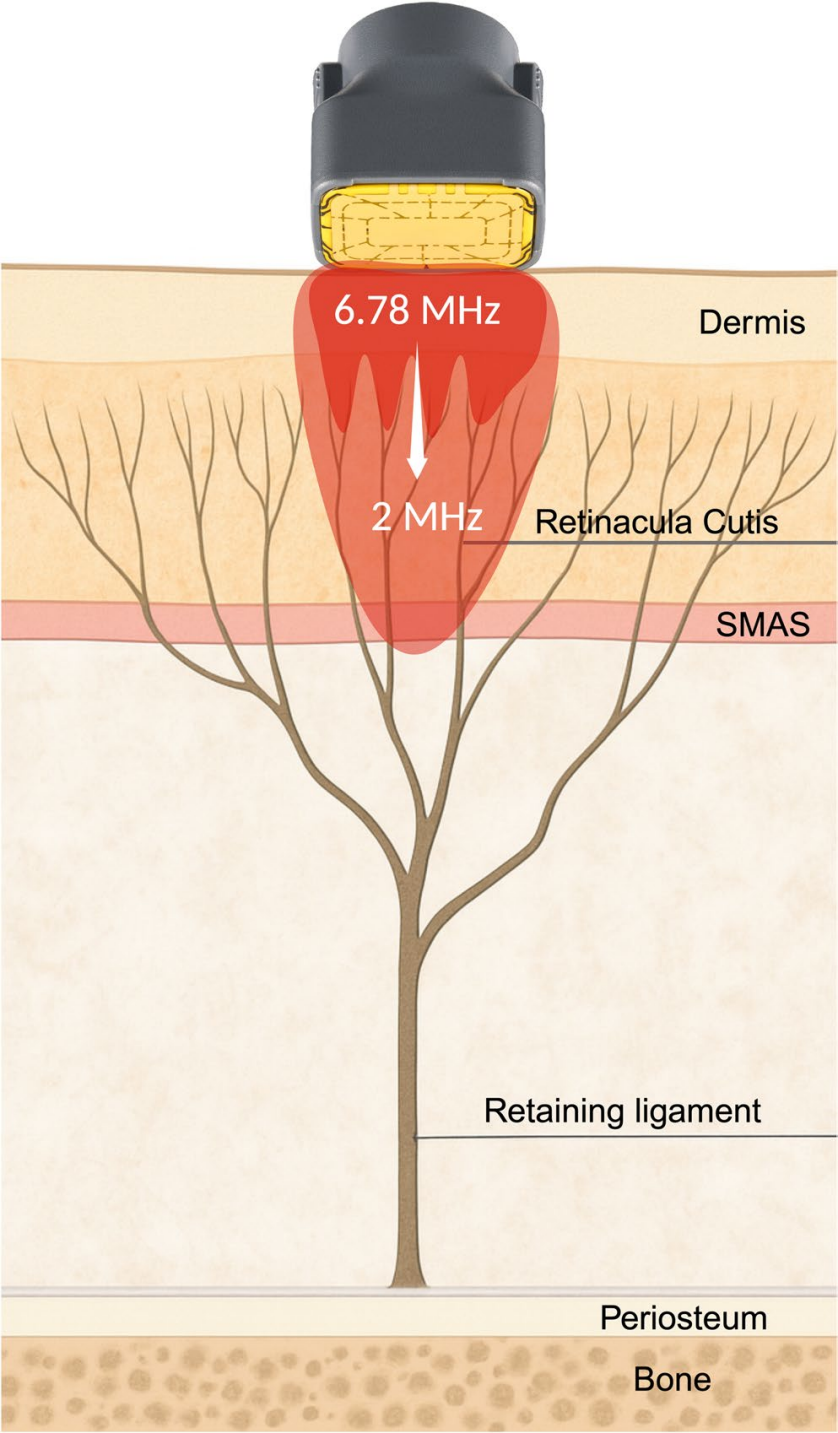
Schematic illustration of the dual-frequency energy distribution of XERF^®^. The shallow mode (6.78 MHz) primarily targets the dermis and retinacula cutis, whereas the deep mode, combining 6.78 MHz and 2 MHz within a single shot, extends its effect to the retinacula cutis, SMAS, and retaining ligaments

The variability in the anatomical depth of NMRF targets across different facial regions, such as the multilayered periorbital SMAS (Fig. 5.) [31–34], can explain the variable outcome of conventional NMRF. XERF controlled multi-depth distributed thermal energy support sustained fibroblast activation, dermal remodeling, and fascial thickening [17, 20]. Our hypothesis is that, by enabling deeper thermal delivery to supportive fascial structures and the multilayered vertical anchoring system (Fig. 6), XERF may overcome regional anatomical variability and enhance tissue-tightening outcomes [18]. We speculate that, when the applicator is continuously moving (i.e., gliding), the specific frequency has only a limited influence on tissue preheating. In contrast, during the stationary stamping phase, sequential delivery of both frequencies to the same treatment location may better exploit their complementary penetration characteristics and facilitate multilayer heating. These proposed mechanism remain speculative but may explain the clinical improvements observed in our study and in previous investigations, together with the high levels of patient satisfaction reported across studies [17–21].

Moreover, XERF integrated cooling and real-time skin temperature monitoring may improve procedural comfort and enhance safety by preventing excessive thermal accumulation, thereby reducing the risk of treatment-related adverse effects such as superficial burns and unintended adipose tissue loss [12, 20].

**Fig. 7:**
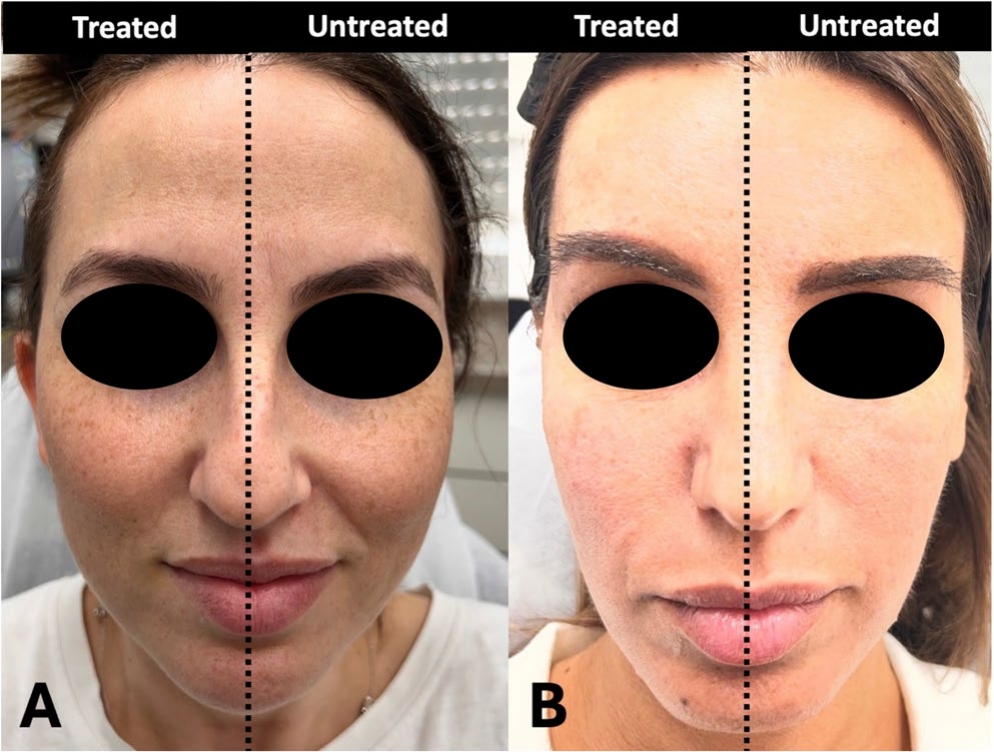
Split-face clinical comparison of immediately post treatment. The dotted midline indicates treated (R. side) versus untreated (L. side) hemiface, allowing intra-individual comparison, in 39-year-old female (**A**) and a 38-year-old female (**B**)

**Fig. 8:**
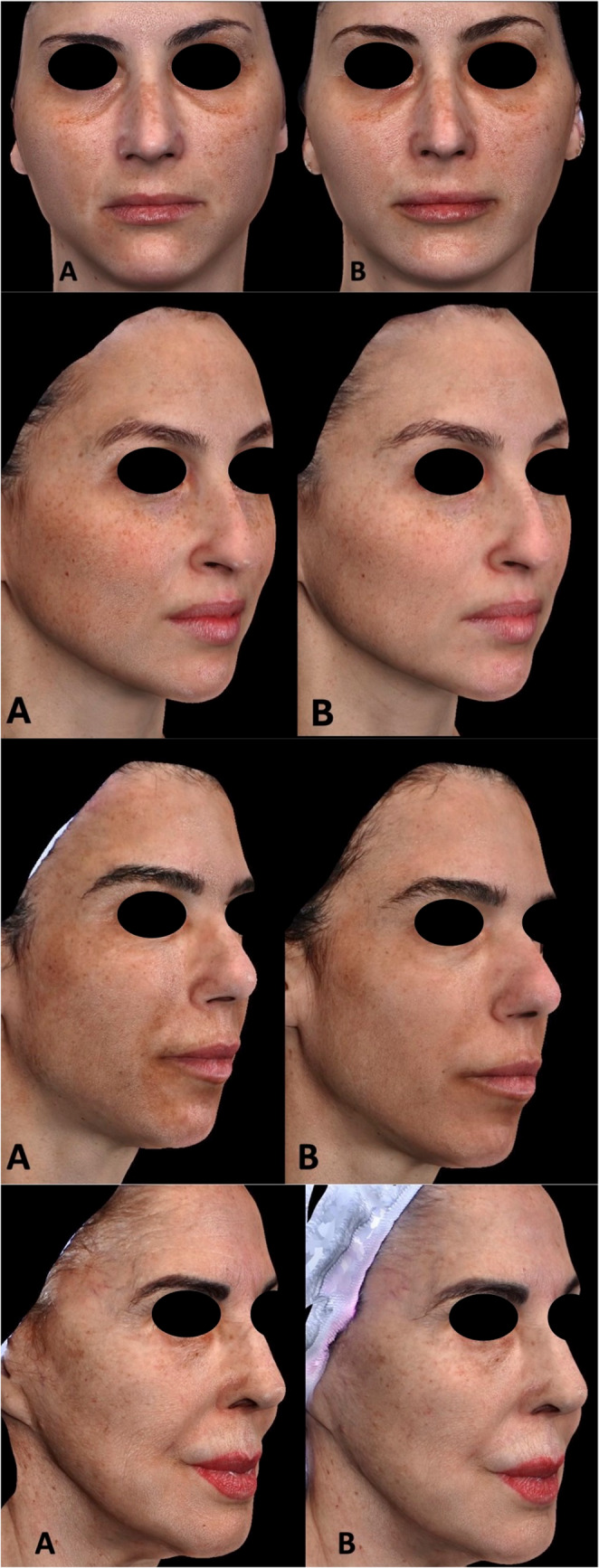
Representative results comparing baseline (**A**) and 3-month follow-up (**B**) obtained using LifeViz^®^ stereophotogrammetry in (**a**) a 38-year-old female, (**b**) a 39-year-old female, (**c**) a 48-year-old female, and (**d**) a 73-year-old female

The present study provides a descriptive evaluation of the effects of XERF on facial skin and soft tissue architecture, integrating objective imaging findings with patient-reported outcomes. A notable observation was a recurring pattern of volumetric redistribution across facial compartments, accompanied by imaging findings suggestive of an immediate lifting and tightening effect (Fig. 7). This pattern may reflect fat-pad repositioning, characterized by an apparent shift from medial to lateral facial volume, with similar findings observed throughout the follow-up period (Fig. 8).

The immediate post-treatment volumetric changes, consisting of lateral volume augmentation and medial volume reduction, may represent an acute tissue response to thermal energy delivery. This early phase may reflect a combination of immediate collagen contraction and transient treatment-induced edema [35]. Although the immediate clinical response does not represent the final treatment outcome, the authors believe it should not be overlooked, as both collagen contraction and edema are biologically meaningful treatment endpoints that may indicate adequate heating of the targeted deep tissues and potentially predict the likelihood of long-term clinical improvement. However, this hypothesis requires further investigation and should be evaluated in prospective studies correlating immediate post-treatment findings with long-term clinical outcomes, which was beyond the scope of the present retrospective analysis.

At the 1-month follow-up, partial regression of the lateral volume increase, together with maintenance of medial volume reduction, may indicate progression from the initial thermal response toward tissue remodeling. These observations are compatible with the expected transition from acute thermal effects to extracellular matrix remodeling and structural reorganization of fascial and ligamentous support tissues. By 3 months, the persistence of medial volume reduction could suggest consolidation of the apparent repositioning effect, consistent with the hypothesis that the early volumetric redistribution may evolve into more stable structural remodeling rather than representing only transient edema [35, 36]. The absence of a significant net volumetric change at 3 months, despite persistent medial volume reduction, is also more compatible with tissue redistribution and structural remodeling than with volume gain or adipose tissue loss, particularly in the absence of any volumizing intervention [37].

Periorbital morphometric analysis demonstrated progressive improvements across all measured parameters, suggesting a possible time-dependent lifting effect. At 3 months, MPBE increased by a mean of + 1.11 mm, lateral canthal brow elevation LCBE by a mean of + 1.44 mm, and SPCE by a mean of + 1.41 mm. Early changes were evident immediately post-treatment, with further improvements observed at 1 and 3 months. Among these parameters, SPCE demonstrated the greatest mean improvement, whereas MPBE and LCBE exhibited greater variability in magnitude and progression. These findings may suggest variable responses of different anatomical subunits within the periorbital region to radiofrequency-induced remodeling, potentially reflecting differences in tissue composition, ligamentous support, and baseline laxity. Notably, the use of smaller applicators may have facilitated more precise and controlled energy delivery across the curved and delicate anatomy of the periorbital region, potentially enabling targeted treatment of fine rhytids and localized laxity while maintaining consistent tissue contact and safety [18].

Objective assessment of skin quality demonstrated progressive improvements in wrinkles, evenness, pore size, and oiliness, with maximal effects observed at 3 months. These findings could indicate that structural and textural skin improvements evolve over time, consistent with a delayed remodeling process. The absence of pigmentation changes in this cohort is reassuring and may support the short-term safety of XERF in patients with pigmentary disorders.

Subjective outcomes were highly concordant with objective findings. Improvements in FACE-Q domains demonstrated statistically significant and clinically meaningful changes in both satisfaction and aging perception. Reductions in perceived age and high levels of overall satisfaction further reinforce the clinical relevance of the observed objective changes. Importantly, all patients expressed willingness to repeat and recommend the treatment, suggesting high acceptability and perceived benefit. This alignment between objective improvements and patient perception is particularly relevant in aesthetic medicine, where patient satisfaction is a key determinant of treatment success.

High patient satisfaction was accompanied by a favorable safety profile, with treatments well tolerated across all facial regions and a mean procedural pain score of 4.31 ± 1.71 on VAS. Treatment-related adverse events were limited to mild and transient reactions, with no significant or persistent complications observed during the follow-up period. Notably, no contour irregularities or unintended volumetric loss were identified, and overall facial volume remained stable aside from the described redistribution patterns.

**Fig. 9:**
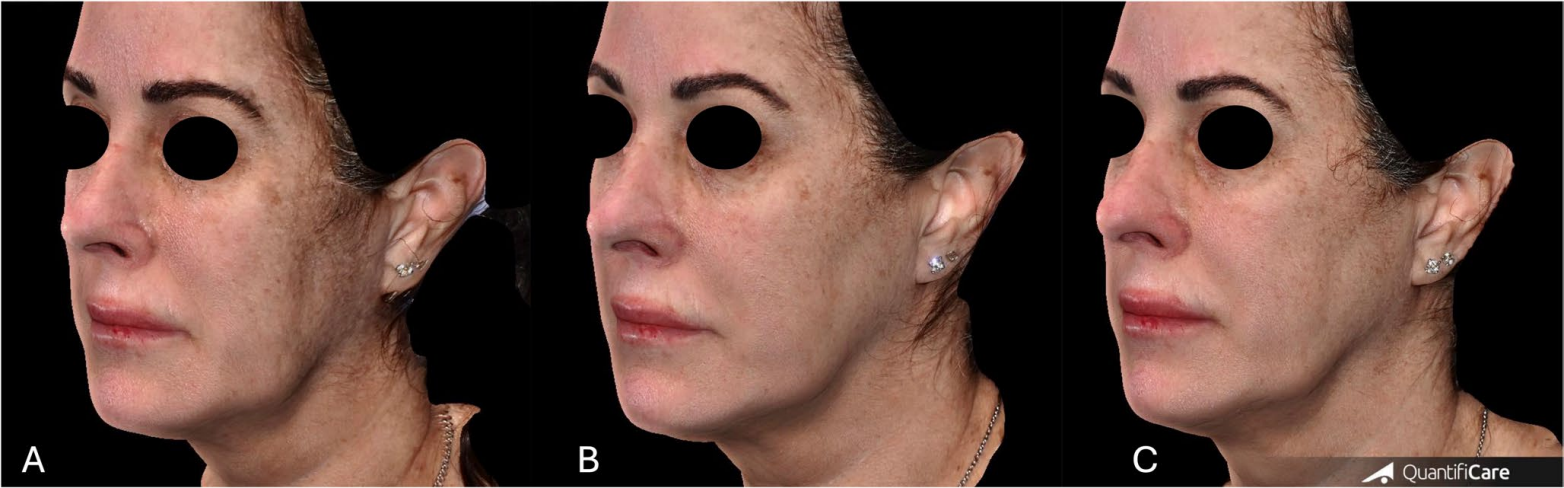
Representative clinical results obtained using LifeViz^®^ stereophotogrammetry in a 60-year-old female, comparing baseline (**A**), 3 months post-treatment (**B**), and 6 months post-treatment (**C**)

The findings of this study should be interpreted in light of several limitations. First, the retrospective design, relatively small sample size, and limited follow-up duration may have influenced the robustness and generalizability of the results and preclude definitive conclusions regarding long-term treatment durability. Although long-term durability cannot be established from the present analysis, during manuscript revision a routine 6-month clinical follow-up became available for a representative patient. Subjectively, the author observed that the clinical improvements appeared to be maintained and, in this individual, may have continued to improve over time (Fig. 9). This observation is presented for illustrative purposes only and was not included in the formal study analysis.

Second, this study did not include a control group or a comparison with other established single-frequency NMRF systems. Therefore, no conclusions can be drawn regarding the superiority of the dual-frequency technology over conventional single-frequency devices.

Third, although patient-reported outcomes were assessed using validated FACE-Q instruments and standardized questionnaires routinely collected in clinical practice, subjective assessments may still be susceptible to response bias inherent to retrospective studies and the absence of blinding.

Finally, the volumetric redistribution methodology applied in this study represents an indirect surrogate of tissue tightening rather than a direct measure of anatomical lifting. This approach has not yet been validated as a standardized outcome metric for skin-tightening interventions. Consequently, the observed shifts in facial volume distribution should be interpreted as suggestive of tissue repositioning rather than definitive evidence of anatomical tissue displacement. Future prospective controlled studies with larger cohorts, longer follow-up periods, and objective assessments of tissue displacement are warranted to further validate these findings.

## Conclusions

The novel dual-frequency single-shot NMRF system, XERF, was associated with favorable clinical outcomes, good treatment tolerability, and no unexpected safety concerns in this retrospective cohort. Improvements in skin tightening and quality were observed using both objective imaging assessments and patient-reported outcomes, accompanied by high levels of patient satisfaction. These preliminary findings suggest that controlled, multilayered thermal energy delivery targeting dermal and fascial structures may contribute to the observed clinical improvements while preserving the structural integrity of the subcutaneous compartment. Larger prospective controlled studies with longer follow-up are warranted to confirm these findings, evaluate their durability, and investigate the underlying mechanisms.

## Data Availability

The datasets generated and/or analyzed during the current study are not publicly available due to patient privacy and confidentiality considerations but are available from the corresponding author on reasonable request and subject to approval by the relevant institutional and ethical requirements.
